# SCN-AVP release of *mPer1/mPer2 *double-mutant mice in vitro

**DOI:** 10.1186/1740-3391-6-5

**Published:** 2008-03-20

**Authors:** Daan R van der Veen, Ellis GA Mulder, Henrik Oster, Menno P Gerkema, Roelof A Hut

**Affiliations:** 1 Department of Chronobiology, University of Groningen, P.O. Box 14, 9750 AA Haren, The Netherlands; 2 Circadian Rhythms Group, Max Planck Institute of Biophysical Chemistry, Am Fassberg 11, 37077 Göttingen, Germany; 3 University of Surrey, Faculty of Health and Medical Sciences, Guildford, Surrey GU2 7XH, UK

## Abstract

**Background:**

Circadian organisation of behavioural and physiological rhythms in mammals is largely driven by the clock in the suprachiasmatic nuclei (SCN) of the hypothalamus. In this clock, a molecular transcriptional repression and activation mechanism generates near 24 hour rhythms. One of the outputs of the molecular clock in specific SCN neurons is arginine-vasopressin (AVP), which is responsive to transcriptional activation by clock gene products. As negative regulators, the protein products of the *period *genes are thought to repress transcriptional activity of the positive limb after heterodimerisation with CRYPTOCHROME. When both the *Per1 *and *Per2 *genes are dysfunctional by targeted deletion of the PAS heterodimer binding domain, mice lose circadian organization of behaviour upon release into constant environmental conditions. To which degree the period genes are involved in the control of AVP output is unknown.

**Methods:**

Using an *in vitro *slice culture setup, SCN-AVP release of cultures made of 10 wildtype and 9 *Per1/2 *double-mutant mice was assayed. Mice were sacrificed in either the early light phase of the light-dark cycle, or in the early subjective day on the first day of constant dark.

**Results:**

Here we report that in arrhythmic homozygous *Per1/2 *double-mutant mice there is still a diurnal peak in *in vitro *AVP release from the SCN similar to that of wildtypes but distinctively different from the release pattern from the paraventricular nucleus. Such a modulation of AVP release is unexpected in mice where the circadian clockwork is thought to be disrupted.

**Conclusion:**

Our results suggest that the circadian clock in these animals, although deficient in (most) behavioural and molecular rhythms, may still be (partially) functional, possibly as an hourglass mechanism. The level of perturbation of the clock in *Per1/2 *double mutants may therefore be less than was originally thought.

## Background

Many behavioural and physiological processes in mammals show circadian (circa 24-hour) rhythms that are entrained to the daily light-dark cycle. These rhythms are governed by internal circadian clocks. The main, light entrainable oscillator is housed in the suprachiasmatic nuclei of the hypothalamus (SCN, [1,2]). As an output of the SCN, Arg^8^-vasopressin (AVP) is expressed predominantly in the dorsomedial subregion of the SCN, also called the *shell *[3,4], and circadian rhythms in SCN-AVP transcription, peptide content and release have been reported [5-10].

Functionally, SCN-AVP content and release have been correlated with variation in behavioural rhythmicity in voles [8,11,12] but cannot be held exclusively responsible for the control of rhythmic organization of behaviour. Brattleboro rats, not expressing functional AVP, still show circadian rhythms in activity – albeit with a decreased amplitude – and entrain to a light dark schedule [13,14]. Species-specific correlation between strength of circadian organization of behaviour and SCN-AVP content [11,15,16] or SCN-AVP release [12] suggest a role for SCN-AVP in the output of the SCN in these species. This correlation between SCN-AVP content/release and behaviour is not necessarily controlled by transcriptional control of the clock [5,17], but can also be a result of posttranscriptional regulation [18]. Whether, and to which degree, components of the circadian clock in the SCN are involved in the transcriptional control of SCN-AVP is the central question of this study. We focus on SCN-AVP release in mice with deletions rendering the *Period1 *and *Period2 *genes dysfunctional, genes that are supposed to be essential parts of the molecular circadian clock in the SCN [19-21].

The presumed pacemaker mechanism in the SCN consists of two interlocking feedback loops of clock gene transcription and translation. In mammals, one loop consists of two transcription factors, *Clock*, being constitutively expressed [22,23] and *Bmal1 *(*Mop3, Arntl*), showing cyclic expression with peak activity in the mid-dark period [24-26]. The protein products CLOCK and BMAL1 form heterodimers which in the nucleus act as e-box binding transcriptional activators [27,28]. In the other loop of the molecular clockwork, the cryptochrome proteins (CRY1 and CRY2) [29,30] strongly inhibit CLOCK:BMAL1 activated transcription [31,32]. The role of the period genes (*Per1, Per2, and Per3 *[33-36]) is more diverse, but each of the *mPer *genes can inhibit CLOCK:BMAL1 activated transcription [5], possibly by heterodimerization to CRY and subsequent translocation into the nucleus, making them part of the negative limb of the feedback loop.

The role of each of the three known *Per *genes has been studied at the behavioural and molecular level separately in corresponding mutant mice. The circadian phenotypes of *mPer *mutants differ for the three period genes. Mice with a mutation in the *mPer1 *gene (either the *mPer1*^*brdm*1 ^mutation ([19], called *Per1*^*m*/*m *^from here on) or an *mPer1*-null mutant [37] show persistent circadian rhythms in constant darkness with a variable, but shorter period than wildtype mice. Mice carrying the *mPer2*^*brdm*1 ^mutation ([20], called *Per2*^*m*/*m *^from here on) exhibit an impaired circadian phenotype. They initially show a circadian rhythm with a short period in DD, but after a few days completely lose circadian rhythmicity in behaviour [20]. Interestingly, under constant illumination levels (LL), both *Per1*^*m*/*m *^and *Per2*^*m*/*m *^show persistent circadian behavioural rhythms, where with increasing light intensity *Per1*^*m*/*m *^mice show increased lengthening of the free-running period length tau and *Per2*^*m*/*m *^mice show shortening of tau [38,39]. Mice with a targeted disruption of the *mPer3 *gene express largely normal circadian rhythms under entrained and free-run conditions, with shorter periods than wildtype animals in DD [40].

Different effects of *Per *mutations are also found at the molecular level. While *mPer1*^*ldc *^mutant animals show no difference from wildtype controls in the expression of *mPer2*, *mCry1 *and *Bmal1 *in the SCN, the amplitude in circadian variation in SCN mPER2 and mCRY1 proteins is markedly reduced [41]. Also in the *Per1*^*m*/*m *^and the *Per1 *null mutant mice no difference in core clock gene expression is seen in the SCN [37,19]. In the periphery, the *Per1 *null mutant (but not the *Per1*^*m*/*m*^) shows prolonged *mPer1 *and *mPer2 *gene expression [37,19]. The impaired behavioural circadian phenotype of the *mPer2*^*ldc *^mutant mice coincides with decreased amplitudes of *mPer1 *and *mCry1 *gene expression in the SCN, whereas *mPer2 *and *Bmal1 *expression are no longer rhythmic. Levels of SCN mPER1 and CRY1 protein are expressed in a circadian rhythm, albeit with a decreased amplitude [41]. Also in the *mPer2*^*m*/*m*^, the amplitude *mPer1 *and *mPer2 *expression is low and the circadian oscillation is lost, and *Bmal1 *levels are truncated and phase advanced, suggesting an additional role for *mPer2 *as a positive regulator of *Bmal1 *[19,42].

*Per1/2 *double-mutant mice lose rhythmicity immediately after release into constant dark [19] as do *mPer1*^*ldc*^*mPer2*^*ldc *^[41] indicating a complete disruption of the clock. Mutants carrying an *mPer3 *mutation and either the *mPer1*^*ldc *^or *mPer2*^*ldc *^mutation do not show circadian phenotypes that are more severe than the single *mPer1*^*ldc *^or *mPer2*^*ldc *^mutation [41]. The behavioural phenotype of the homozygous *Per1*^*m*/*m*^*Per2*^*m*/*m *^double-mutant mice suggests that both genes are essential for the generation of circadian rhythms and cannot be substituted by *Per3 *while a certain degree of redundancy between both *Per1 *and *Per2 *is apparent [19].

When the double mutation in *Per1 *and *Per2 *is combined with a mutation in one of the binding partners *Cry1 *or *Cry2 *to form triple knock out animals, again an arrhythmic behavioural phenotype is seen [43]. In these animals, all rhythms in SCN-AVP are lost. To address the question whether post transcriptional regulation of SCN-AVP depends on the presence of the *Per1 *and *Per2 *genes we used a fully automated acute slice culture system to measure SCN and paraventricular AVP release in brain slices from homozygous *Per1*^*m*/*m*^*Per2*^*m*/*m *^mutants.

## Materials and Methods

### Animals

Mice homozygous for both the *mPer1*^*m*/*m *^and *mPer2*^*m*/*m *^mutation ([19-21], from here onwards denoted as *Per1*^*m*/*m*^*Per2*^*m*/*m*^) (N = 10) and congenic wildtype mice (N = 9), derived from a C57Bl/6 × 129/sv mixed background stock originally received from U. Albrecht, were bred in our mouse facility in Haren, The Netherlands. Adult male *Per1*^*m*/*m*^*Per2*^*m*/*m *^homozygote double-mutant mice and wildtype mice originating from *Per1*^*m*/+ ^× *Per1*^*m*/+ ^and a *Per2*^*m*/+ ^× *Per2*^*m*/+ ^breeding lines were housed individually in translucent cages (Macrolon type 1 long) equipped with a running wheel (∅14 cm) and passive infrared detection (PIR) on the cage lids. Prior to entrainment to a LD schedule, *Per1*^*m*/*m*^*Per2*^*m*/*m *^mutant mice were kept in constant darkness for two weeks. Activity measurements, binned in two minute intervals, were recorded on our Event Recording System (ERS) and stored for behavioural analysis. Food and tap water were available *ad libitum *and cages were placed in a sound attenuated, temperature controlled room (22 ± 0.5°C; 60% humidity; light intensity 250–350 lux, depending on cage placement).

Mice were entrained to a 14 hrs light: 10 hrs dark cycle (LD 14:10) for at least 2 weeks before being sacrificed for culturing. Before the onset of the experiment, mice either remained under LD 14:10 or lights were not turned on at the last morning. For culturing of SCN tissue, mice were sacrificed in the first half of the light period (External Time 9.9 ± 0.7 (SEM), ExT; [44], N = 13) or in the first day of dark in the first half of the subjective day (Internal Time 8.3 ± 0.2 (SEM), InT, N = 7). All experiments were approved by the Animal Experimentation Committee of the University of Groningen (DEC No. 2595).

### Culturing

Animals were deeply anesthetized by inhalation of isoflurane (Forene, Abbott laboratories) and transferred to a 'clean room', with a slight overpressure and HEPA filtered air flow. Animals were then briefly dipped in 70% ethanol and immediately decapitated. The whole brain was dissected out and rinsed in ice-cooled, sterile and oxygenated Gey's balanced salt solution for two minutes. Brains were trimmed down to a block containing the hypothalamus using a handheld scalpel. After trimming a hand operated tissue chopper (tissue slicer 51425, Stoelting, Illinois, USA) was used to cut coronal sections of 300 μm, containing the SCN. Under a dissection microscope a section containing the mid-part of the SCN was selected and was trimmed to the height and width of the two SCN nuclei, leaving a bilateral SCN slice explant including the optic chiasm of approximately 1 by 1 mm and a thickness of 300 μm. For two *Per1*^*m*/*m*^*Per2*^*m*/*m *^mutant mice (one sacrificed in LD and one sacrificed in DD) similar sized sections of the paraventricular nucleus of the hypothalamus (PVN) were made. All sections were stored in oxygenized Gey's balanced salt solution for 2 to 3 hours, at 4°C.

Acute slice cultures were prepared in a small culture well in our custom built automated culture system with a continuous medium flow kept in a laminar flow cabinet. In brief, cultures were placed in a small culturing well milled out of anodized aluminium which was continuously kept at a temperature of 37 ± 0.26°C and was filled with carbogenated Earle's balanced salt solution with NaHCO_3 _(Sigma-Aldrich; with added antifungal antibiotic Amphotericin B (2 mg/l)). When all cultures were placed, the lid was mounted, closing off all the individual chambers from each other and the outside environment, except for a decompression tube leading into a small flow of carbogen, making sure that the system was not pressurized. Upon closing, the medium was flushed through the wells at a speed of 325 μl/hour per well. Well volume was 160 μl, thus at these perfusion rates the exchange rate was ~2× per hour. Cultures remained stationary in the well and were submersed in the medium. The medium outflow containing the release products of the culture of the individual wells was collected immediately at -40°C, starting a new collection every 2 hours. Samples were kept at -40°C until further analysis.

### Radio Immuno Assay

For the Radio Immuno Assay a standard kit by Euro-Diagnostica (Mediphos) was used. In short, all samples were analyzed in duplicate and a standard dilution curves ranging from 0.47 to 60 pmol/liter (corresponding to an *in vitro *SCN-AVP release of 0.329 – 42 fmol/2 hours), a low and a high control were included in every assay. The assay applies an ^125^I-labelled AVP tracer (1700 – 2100 μCi/nmol) as readout, with primary antibody rabbit anti-AVP which is precipitated by a solid phase secondary antibody (goat anti-rabbit IgG) bound to cellulose. Individual values of AVP release were expressed as fmol/2 hours.

Using circular statistics, individual release profiles were checked for rhythmicity using a harmonic regression analysis (2 harmonics, CircWave [45]). Grouped release data was represented as percentage of average. Peaks in AVP release were identified using t-tests for each time point (a modified 'least significant difference' (LSD) method), comparing average values for each time point to the average level of the group throughout the time series using a significance level of 0.05. As a control, data points were randomly selected from the dataset and analysed similarly as the release data.

## Results

**Figure 1:**
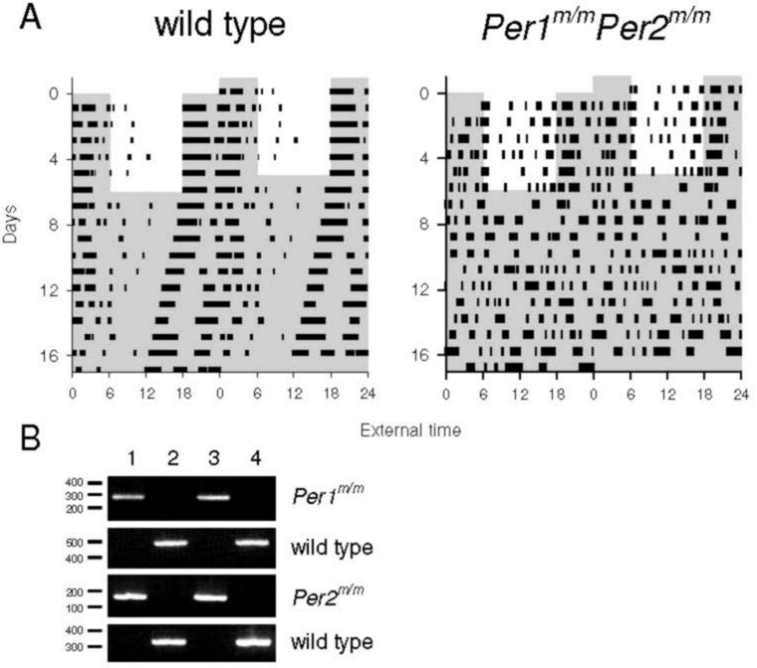
**Activity patterns and genotypes**. A) Double plotted actogram of a wildtype mouse (left panel) and a *Per1*^*m*/*m*^*Per2*^*m*/*m *^double-mutant mouse (right panel) during a period of light-dark 12:12 followed by a free running rhythms in continuous dim red light. B) Genotyping example of four animals used in this study. Using the same primer sets, lane 1 and 3 show the lighter PCR products for the *Per1 *and the *Per2 *fragments indicating the *Per1*^*m*/*m*^*Per2*^*m*/*m *^double-mutant genotype in both mice, lane 2 and 4 show the heavier PCR products for both genes indicating the *Per1*^+/+^*Per2*^+/+ ^genotype in both mice. Molecular weight scales are in kiloDalton.

In our hands *Per1*^*m*/*m*^*Per2*^*m*/*m *^double-mutant mice are arrhythmic whereas wildtype mice show robust free-running circadian rhythms in DD with a period of 23.72 hrs (SEM = 0.06 hrs, N = 12, [46]). For the *Per1*^*m*/*m*^*Per2*^*m*/*m *^double-mutant mice, behavioural arrhythmicity in DD is confirmed by the rhythmicity index (maximal ΔqP ± SEM within 20–28 hrs, [46]) which was negative (-180.25 ± 18.12, N = 12), while the robust behavioural rhythms of wildtype mice have maximal values of 2507.35 ± 193.10 at their respective periods of free-running rhythms in DD(N = 12) (see Fig. 1).

**Figure 2:**
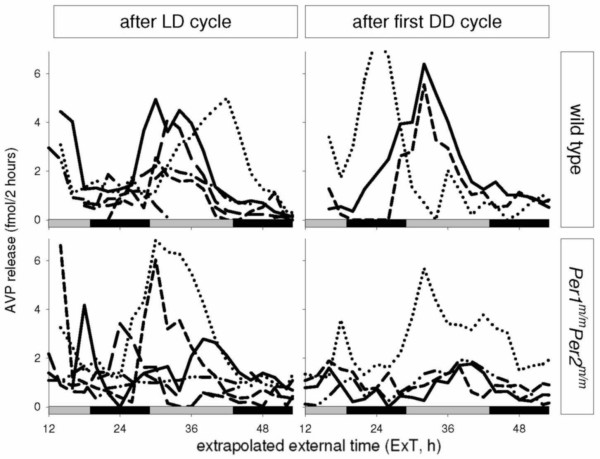
**Individual release patterns of AVP from *in vitro *SCN slice cultures**. Slice cultures were made from wildtype (upper panels) and *Per1*^*m*/*m*^*Per2*^*m*/*m *^double-mutant mice (lower panels). Release pattern is shown as a function of external time, extrapolated from time of sacrifice. Right panels show release patterns of mice sacrificed during LD, and left panels show release patterns of mice sacrificed during the first day of DD. All cultures showed significant circadian rhythmicity in AVP release pattern with periods between 18–32 h, between ExT 10–44 h (except one culture from WT mice under LD). Different line types indicate SCN-AVP release of an individual culture. Black/grey bars indicate projected night and day, respectively.

Average SCN-AVP release during the first 24 hours (± SD, maximum release) was assayed at 2.61 (± 1.63, max = 13.04) fmol/2 hours for wildtype and 1.87 (± .06, max = 6.86) fmol/2 hours for *Per1*^*m*/*m*^*Per2*^*m*/*m *^mutant mice. Levels of release did not differ between wildtypes and *Per1*^*m*/*m*^*Per2*^*m*/*m *^mutant mice, between mice sacrificed in LD or DD nor was there an interaction effect of genotype and time of sacrifice on overall levels of release (two-way ANOVA; P's > 0.05). Individual SCN-AVP release profiles are shown in Figure 2. For both the wildtype and *Per1*^*m*/*m*^*Per2*^*m*/*m *^mutant mice the individual curves in LD show strong variation over time with similar peak levels. All individual SCN cultures showed significant circadian rhythmicity in the release pattern between ExT 10–44 h (except one culture from WT mice under LD, which suffered from missing data). The detected rhythmicity in the circadian domain with periods between 18–32 h (p < 0.015 corrected for multiple period testing (CircWave [45], Harmonic regression with two harmonics).

For mice sacrificed in DD peak SCN-AVP release levels within individual *Per1*^*m*/*m*^*Per2*^*m*/*m *^mice seem to vary more in phase and amplitude than wildtype mice. The slope of linear decline during the session was on average -0.046 ± 0.027 for wildtypes and -0.027 ± 0.019 for *Per1*^*m*/*m*^*Per2*^*m*/*m *^mutant mice, indicating that the variability of both cultures was similar (t-test, P > 0.05).

**Figure 3:**
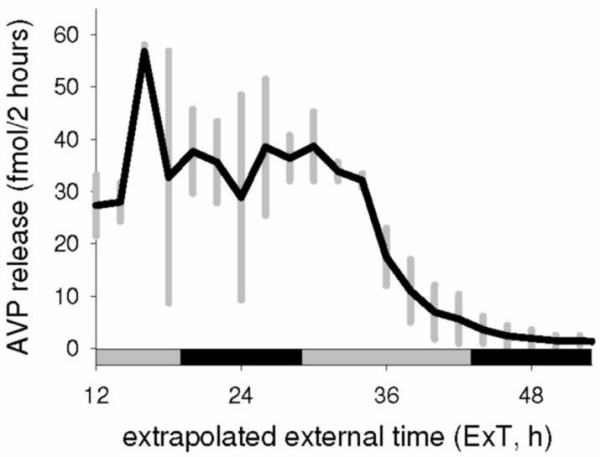
**Paraventricular AVP release**. Shown is the average spontaneous paraventricular AVP release of 2 SCN cultures made from *Per1*^*m*/*m*^*Per2*^*m*/*m *^double-mutant mice (one sacrificed in LD and one in DD). Paraventricular release is different from that of the SCN in both quantity and shape.

As a control, paraventricular AVP release of *Per1*^*m*/*m*^*Per2*^*m*/*m *^mutant mice was measured, shown in Figure 3. Release was constantly high at the beginning of the culturing with no detectable circadian fluctuation, but decreased after time (slopes are -1.2 and -0.8 for PVN cultures taken from DD and LD respectively). Average paraventricular AVP release of the first 24 hours was 35.56 (± 13.31) fmol/2 hours, with a maximal value of 58.03 fmol/2 hours. SCN-AVP release was significantly lower than paraventricular AVP release (Mann-Whitney Rank Sum Test, P < 0.001).

**Figure 4:**
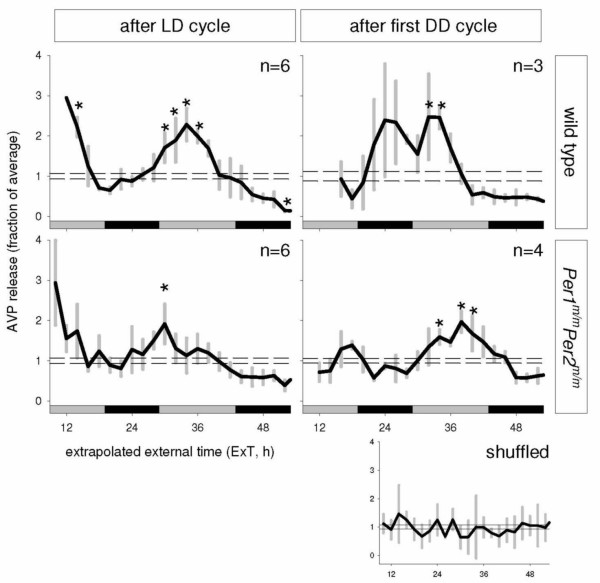
**Average SCN-AVP release patterns for cultures made from wildtype (upper panels) and *Per1*^*m*/*m*^*Per2*^*m*/*m *^double-mutant mice (lower panels)**. Percent of average release and SEM are plotted in external time, extrapolated from the time of sacrifice. Left panels show release patterns of mice sacrificed during LD, right panels show release patterns of mice sacrificed during the first day of DD. The very bottom right panel shows the profile of randomly selected samples. Asterisks indicate samples with a release level significantly deviating from the average (modified LSD test; P < 0.05). Horizontal dashed lines depict the SEMs. Black/grey bars indicate projected night and day, respectively.

The two left panels in Figure 4 show average SCN-AVP release profiles of cultures made from wildtype and *Per1*^*m*/*m*^*Per2*^*m*/*m *^mutant mice sacrificed in the first half of the light period. After a decline in SCN-AVP release in the first hours of culturing, levels increased to peak levels in the beginning of the extrapolated day and subsequently decreased again. Comparing the release levels of individual time points to culturing average indicate significantly elevated diurnal levels of SCN-AVP release in the extrapolated external (ExT) 6–12 for wildtypes and ExT 6 for *Per1*^*m*/*m*^*Per2*^*m*/*m *^(Least square difference (LSD) test); P's < 0.01). Although the diurnal peak in SCN-AVP release in cultures made of *Per1*^*m*/*m*^*Per2*^*m*/*m *^mice was less distinct, the timing was similar to that of the wildtype controls.

In Figure 4, the right panels show average release profiles of SCN-AVP of cultures made of wildtype and *Per1*^*m*/*m*^*Per2*^*m*/*m *^mutant animals sacrificed on the first day of DD. SCN-AVP release profiles did not show a marked initial high level of SCN-AVP release as did those of animals sacrificed in LD. Both cultures made of wildtype and *Per1*^*m*/*m*^*Per2*^*m*/*m *^mutant animals showed increasing levels of SCN-AVP release peaking in the extrapolated day. The LSD test against the average level of each group indicates significant increased levels at ExT 8 and 10 for wildtypes and ExT 10, 14 and 16 for *Per1*^*m*/*m*^*Per2*^*m*/*m *^mutants, which is later than for the animals sacrificed in LD.

The diurnal peak in SCN-AVP expression in cultures made from wildtype mice shows similar peak phasing in animals sacrificed in LD (ExT 6–12) and animals sacrificed in DD (ExT 8–10). SCN-AVP release in cultures made from *Per1*^*m*/*m*^*Per2*^*m*/*m *^shows a peak release with a similar phase compared to wildtype when sacrificed in LD (ExT 6), but is delayed when animals are sacrificed in DD (ExT 10, 14–16), indicative for a longer inter-peak interval or period.

SCN-AVP release values were randomly selected out of all release data and averaged in 2-h bins (Fig. 4, "shuffled"). The pattern through these points is different from that of the release profiles and essentially flat. Using the same statistical method that was used to identify significantly elevated levels of SCN-AVP release in the cultures does not identify any significant changes in AVP levels for any data point.

## Discussion

In our *in vitro *culture system both cultures made of wildtype and *Per1*^*m*/*m*^*Per2*^*m*/*m *^double-mutant mice show rhythmic AVP release patterns with peak secretion of AVP well after onset of culturing. Although our data do not conclusively indicate rhythmicity in the *in vitro *release of SCN-AVP in wildtype and *Per1*^*m*/*m*^*Per2*^*m*/*m *^double-mutant mice – because of the lack of multiple cycles – the patterns of SCN-AVP release do fluctuate over time as would be predicted from SCN-AVP release *in *v*itro *[9,10]. Moreover, the data indicate that this fluctuation does not differ between wildtype and *Per1*^*m*/*m*^*Per2*^*m*/*m *^double-mutant mice. The attenuation of PVN-AVP release over time indicates that in our culturing method, no evidence of multiple circadian cycles in AVP release can be seen, we can therefore not distinguish between a circadian and an hourglass process. The flat and dissimilar profiles seen when the dataset is randomly sampled and the marked differences between SCN- and PVN-AVP release profiles in our data do however suggest a non random SCN-AVP release with peak release levels that are similarly timed between wildtype and *Per1*^*m*/*m*^*Per2*^*m*/*m *^double-mutant mice, but are not seen in the gradually decreasing levels of PVN-AVP release. While individual curves of *Per1*^*m*/*m*^*Per2*^*m*/*m *^double-mutant mice sacrificed in DD do suggest greater variation in phase and amplitude within individual, correcting for these individual amplitude differences through expressing average profiles as percent of average release, rather than absolute release shows clear peaked release. It is not unlikely that the loss of the *Per1 *and *Per2 *gene may be involved in the greater variance of AVP release in the SCN cultures of these individuals, but such impairment does not lead to loss of peaks in release.

Mice carrying both the *Per1*^*m*/*m *^and the *Per2*^*m*/*m *^mutations show an arrhythmic circadian phenotype [19]. Core clock gene expression is severely impaired in these mice, indicative of a disrupted circadian clock. A similar SCN-AVP release between wildtype and *Per1*^*m*/*m*^*Per2*^*m*/*m *^double-mutant mice may be indicative for a partially intact clock mechanism. Remaining functionality could possibly include a strongly damped oscillator or an hour glass mechanism, but our data do not prove any residual oscillator function. In SCN slices of m*Cry1/mCry2 *double-mutant mice (also carrying a disrupted clock), a single peak in electrical activity has been reported [47]. This peak was only seen when the animals were sacrificed at the beginning of the light period, but not when they were sacrificed at the beginning of dark. The authors hypothesized that the absence of such a peak in electrical activity in slices of animals sacrificed at the beginning of the dark might result from the fact that activity is induced by the light exposure and not by the culturing technique. In our case, release of SCN-AVP induced directly by light is less likely as both animals sacrificed in the light phase of the LD cycle and animals sacrificed in the dark on the first day in DD showed a peak in SCN-AVP release. If, in our case, an hourglass mechanism underlies our *ex vivo *SCN-AVP release, it cannot be driven by light. However, we cannot completely exclude other unknown factors related to our culturing technique, which might drive the observed peaks in AVP release.

It is known that serum in the medium of tissue cultures can affect cellular rhythmicity. In cultured fibroblasts, circadian rhythms are induced (possibly due to synchronization of the single cells) and reset by serum shock [48]. More recently, fibroblast cultures of multiple clock gene *knock out *mice including *Cry1*^-/-^*Cry2*^-/- ^have been shown to respond to a medium change by increased *Per2 *expression [49]. One of the mechanisms by which this can be achieved is through the nuclear localization of mPER proteins in response to an unknown serum signal [50]. A one-time induction of the negative limb of the clock (possibly through residual functionality of *Per1*^*m*/*m *^and *Per2*^*m*/*m *^products, or the intact *Per3 *gene) could result in one (partial) oscillation resulting in an hour glass-like effect.

As mentioned above, light does not seem to turn on this hour glass, but some phasing effects of light in release patterns of SCN-AVP in our cultures may be present. Although the peak of SCN-AVP release of wildtype mice is less pronounced in DD than in LD, the timing of the peak of AVP release of wildtype mice in LD and DD does not suggest being very different. From our data we can however not clearly establish a phase or period in SCN-AVP release in cultures from wildtype mice from DD. In *Per1*^*m*/*m*^*Per2*^*m*/*m *^double-mutant mice, the timing of the peak in SCN-AVP release in DD is markedly later than in LD. While *in vivo *these animals are arrhythmic in DD and the clock is perturbed, this delayed peak in DD in comparison to LD could be indicative of a long intrinsic period, only apparent under specific circumstances.

The extent to which the circadian clock is disabled by *knocking out Period *genes is unclear. Xu *et al. *[51] reported that when the *Per2*^*ldc *^mutation was crossed into a C57B/6J background (different from the original 129/sv background in [41]), the behavioural phenotype of a short tau and eventual circadian arrhythmicity in DD is lost and a wildtype phenotype is rescued. The level of perturbation of the clock by *Period *deletion thus seems highly dependant on the genetic background. Our findings support this view of a (partially) functional clock. While not conclusive, the data presented here on timed peaks in *in vitro *SCN-AVP release in *Per1*^*m*/*m*^*Per2*^*m*/*m *^double mutants raise the possibility of partial preservation of a clock function in these mice. They might raise questions to what extend the clock function is disabled in *Per1*^*m*/*m*^*Per2*^*m*/*m *^double mutants.

## List of abbreviations

AVP: Arg^8^-vasopressin; ExT: external time; LSD: least significant difference; PVN: paraventricular nucleus of the hypothalamus; SCN: suprachiasmatic nuclei of the hypothalamus.

## Competing interests

The author(s) declare that they have no competing interests.

## Authors' contributions

DRvdV, MPG and RAH designed the study. DRvdV, GAM and RAH collected data, performed RIA and processed the data. HO performed genotyping and contributed to the manuscript. DRvdV and RAH analysed data. DRvdV wrote the manuscript. All authors read and approved the final manuscript.
